# Lectures on Clinical Uses of Electricity

**Published:** 1874-07

**Authors:** 


### Books Reviewed.

*Lectures on the Clinical Uses of Electricity.* Delivered in University College Hospital. By J. Russell Reynolds, M. D., F. R. S., etc. Second Edition. Philadelphia: Lindsay & Blakiston, 1874.

*Galvano-Therapeutics.* A Revised reprint of a Report made to the Illinois State Medical Society, 1873. Philadelphia: Lindsay & Blakiston, 1873.

Dr. Reynold's lectures upon the Clinical Uses of Electricity is one of the best manuals of the subject with which we are acquainted. Avoiding the technicalities and debatable points of the science, he introduces his hearer at once by plain and simple explanations to a full view of the subject. One of the obstacles to the study of this subject is, that so few text books or manuals are found which are free from the constant use of terms understood alone by the author, or from long discussions of undetermined points in Electro-Physiology. We are glad that a new Edition has been placed before the profession and can cordially recommend it to their perusal.

